# Progressive Degeneracy

**Published:** 1912-08

**Authors:** 


### Progressive Degeneracy.

*"What a falling off was there, my countrymen!"*

Is there a worm in the bud,—a fly in the ointment—a Senegambian in the fuel—or, what *is* the matter with the Octopus,—the great A. M. A.?

*American Medicine* (June) in a lengthy editorial commenting on the recent meeting at Atlantic City says:

"The Secretary in his report gave the following: 'The membership of the American Medical Association on May 1, 1911, was 33,960. During the past year, 299 members have died, 1301 have resigned, 500 have been dropped as not eligible, 987 have been dropped for non-payment of dues and 64 have been removed from the rolls on account of being reported "not found," making a total of 3151 names to be deducted from the membership list. There have been added 3474 names to the membership roll, of which 2353 were transferred from the subscription list. The membership of the American Medical Association on May 1, 1912, was 34,283, a net increase for the year of 323.'

"In other words, but for the transfer of names from the subscription list to the membership list *there would have been an actual loss of 2030 members!* The net increase of 323 referred to in the Secretary's report—eliminating the fact that it was made possible by the transfer of 2353 names from the subscription list—is a pitifully small gain when it is remembered that on May 1, 1911, there were at least 70,000 physicians not affiliated with the Association who were eligible to membership. Looked at from every angle this condition is not a healthy one. Something is to blame not only for the fact that 90 per cent of the members fail to attend the annual meeting—even when it is held in the most desirable place in the country—but also that over two-thirds of the eligible physicians of the United States can not be induced to become members of the Association."

Thirteen hundred resign! Nearly one thousand refuse to *ante* (which is equivalent to resigning), making nearly 2300 who go out, not counting dead and ineligibles. It seems that there were 2353 subscribers to the *Journal* who were not members of the

Association, although membership costs nothing, but *goes* with each $5.00 subscription, but these fellows didn't want it. To make a show and partly fill the gap, these are dragged in, and their names put on the roll—a regular rape, and yet, in summing up, the report shows a net gain of 323!

What's the matter? Why this falling off, my countrymen? A few years ago seven thousand or eight thousand members registered at a Boston or Chicago meeting; now barely three thousand attend at the most delightful summer resort at the most delightful season of the year.

It reminds me of the convalescent who fell off until he was so thin that he had to put a rock in each pocket in order to *weigh anything at all!*

At this rate of falling off there will soon be a shadow or skeleton of a once proud and popular organization. I ask again, What's wrong? Is it a case of progressive degeneracy from inherent causes?—bad management, or is it too much Simmons and Council on Pharmacy? Seventy odd thousand eligible American physicians decline to become a part of the dog, to be wagged by such a tail!

